# Full-scale walk-in containerized lithium-ion battery energy storage system fire test data

**DOI:** 10.1016/j.dib.2022.108712

**Published:** 2022-10-29

**Authors:** Mark McKinnon, Adam Barowy, Alexandra Schraiber, Jack Regan

**Affiliations:** a UL's Fire Safety Research Institute, 6200 Old Dobbin Ln. Ste. 150 Columbia, MD 21045, United States; b UL Solutions Fire Research and Development, 333 Pfingsten Road, Northbrook, IL 60062, United States

**Keywords:** Lithium-Ion, Thermal runaway, Energy storage system, Fire, Suppression, UL 9540A Test method

## Abstract

Three installation-level lithium-ion battery (LIB) energy storage system (ESS) tests were conducted to the specifications of the UL 9540A standard test method [1]. Each test included a mocked-up initiating ESS unit rack and two target ESS unit racks installed within a standard size 6.06 m (20 ft) International Organization for Standardization (ISO) container. All tests were conducted with an identical LIB configuration. The initiating unit rack included nine modules (2,430 individual 18650 form factor cells) with a total capacity of 28.9 kWh. The target unit racks were loaded to one-third capacity of the initiating unit with nine partial modules and a total capacity of 9.6 kWh. All cells in the container were charged to 100% state-of-charge and none were electrically connected. Within the initiating mock-up unit, a flexible film heater was wrapped around an individual 18650 form factor cell. This instrumented 18650 cell was heated at a rate of 6°C/min to initiate thermal runaway.

Test 1 was a baseline performance test and did not utilize any active fire suppression systems. Test 2 included a Novec 1230 system designed for an 8.3 vol% concentration discharged upon activation of two smoke detectors installed inside the container. Test 3 incorporated a dry pipe water suppression system to provide a uniform 20.8 mm/min (0.5 gpm/ft^2^) spray density delivered at the top of the ESS unit enclosures.

Thermocouples were used to measure the cell temperatures in the initiating unit rack and module surface temperatures for the initiating unit and target unit racks. Thermocouples were located throughout the ISO container to measure gas temperatures and wall temperatures. Schmidt-Boelter heat flux gauges were installed to measure incident heat flux to each of the target unit racks as well as the walls adjacent to the initiating rack. Smoke detectors and smoke obscuration meters were used to identify the presence of smoke and characterize opacity of the smoke in the container. Various laboratory- and industrial-grade sensors were used to characterize the gas composition throughout container.

**Specifications Table**

**Table:** 

Subject	Engineering
Specific subject area	Experimental Thermal and Fluid Sciences, Fire Safety Engineering, Lithium Ion Battery Energy Storage System Thermal Runaway Thermal and Chemical Characterization
Type of data	Table
How the data were acquired	Data were collected via sensors which included the following: • Type K Thermocouples • Medtherm 64SB20 Schmidt-Boelter Heat Flux Gauges • Siemens Oxymat 6E Paramagnetic Oxygen Sensor • Siemens Ultramat 23 Nondispersive Infrared Carbon dioxide/Carbon monoxide Sensor • Intec Controls SPC31112 Electrochemical Carbon monoxide Sensor • Hy-Optima 2720 Palladium-Nickel Hydrogen Sensor • GDS GasMax II-05-014-20/00-000-00/0-0-SS Electrochemical Hydrogen Sensor • J.U.M. 3-300A/OVE Flame Ionization Detector • J.U.M. 3-500 Flame Ionization Detector • Macurco GD-12 Catalytic Bead Combustible Gas Sensor • Smoke Obscuration Meter using a Huygen Photometer • Kidde i9070 Smoke Detector A National Instruments SCXI-1001 chassis, SCXI-1600 DAQ controller, SCXI-1102 voltage input multiplexer, and a SCXI-TC2095 thermocouple input module were used to collect the data from the listed sensors. A custom National Instruments LabView VI was built to acquire and log the data.
Data format	Raw
Description of data collection	One cell level lithium-ion battery (LIB) and three installation level LIB energy storage system (ESS) tests were conducted in general accordance with the UL 9540A Test Method [1]. The cell level test involved a mock-up cell with thirty 18650 form factor LIB cells. A single 18650 cell was forced into thermal runaway to begin propagating thermal runaway through the mock-up cell. Each installation level test included a mock-up initiating unit rack and two target unit racks installed within a standard size 6.06 m (20 ft) International Organization for Standardization (ISO) container. The initiating unit was forced into thermal runaway and the resulting thermal conditions and chemical concentrations in the container were measured.
Data source location	• Institution: UL • City/Town/Region: Northbrook, Illinois • Country: United States • Latitude and longitude for collected samples/data:42.14616, -87.84650
Data accessibility	The raw data described in this work are archived in the following: Repository name: Zenodo Data identification number: https://doi.org/10.5281/zenodo.7003380[3] Repository name: GitHub Direct URL to the data: https://github.com/ulfsri/fsri-lib-ess-demo-2020

## Value of the Data

• These data demonstrate the thermal and chemical conditions generated within an installation-level ESS during a propagating thermal runaway event and the effect of common fire suppression techniques on those conditions. These data typically remain confidential with ESS manufacturers and integrators after testing, which hinders progress in safe design of such systems.
• These data may benefit engineers and designers of ESS and other systems that utilize LIBs, researchers exploring optimization of LIB system and LIB ESS safety, and developers and practitioners of fire safety models and deflagration models seeking model validation data.
• These data may be used to analyze the thermal load from typical LIB units to surroundings, the gas composition of an enclosed space in which thermal runaway is progressing, and the effectiveness of detection and fire suppressive measures for LIB arrays for safety system design and validation.
• These data may be used by LIB system designers and integrators to inform the next generation of system designs.
• These data may inform first responders, authorities having jurisdiction (AHJ) and building inspectors as to the possible hazards associated with LIBs undergoing thermal runaway and the efficacy of state-of-the-art detection and suppression techniques.

## 1 Objective

Lithium-ion battery (LIB) energy storage systems (ESS) are an essential component of a sustainable and resilient modern electrical grid. ESS allow for power stability during increasing strain on the grid and a global push toward an increased reliance on intermittent renewable energy sources. LIBs are the most economical storage medium currently available for ESS, but inherent in the design and chemistry of LIBs is the potential for a rapid exothermic reaction called thermal runaway. Thermal runaway can propagate through battery arrays and result in the release of thermal energy as well as toxic and flammable gases and vapors that can create explosion hazards. With this relatively new technology undergoing rapid development and adoption, private companies have invested significantly in refining proprietary designs for ESS and protective measures. The data collected in early experimentation with these systems has remained proprietary and confidential and the high cost of this experimentation has led to insurmountable barriers to access for researchers, product developers, and public safety officials and, in effect, reduced awareness and safety of the public. Because of these barriers, no data on thermal runaway at the installation-level scale have been published before. The authors strive to eliminate the barriers to access to data on LIB ESS and the effectiveness of detection and suppression measures commonly installed in containerized ESS.

## 2 Data Description

### 2.1 Data description

The github repository contains the data and supporting files from one cell-level mock-up experiment and three installation-scale lithium-ion battery (LIB) energy storage system (ESS) mock-up experiments conducted in accordance with the UL 9540A Standard Test Method [1]. The repository contains directories for the raw data and event timestamps and supporting information for the experiments.

### 2.2 Data

The Data directory contains a subdirectory for each of the experiments. All data files are in comma-separated value (CSV) plain text format. The data files include time series data collected for all sensors and detectors used to instrument the mock-up cell and simulated ESS. The Data directory also includes a CSV file that details the timestamp for each significant event in the experiment timeline. All data from each experiment were collected and have been stored at a frequency of 1 Hz.

Thermocouple data are presented in the provided tables in°C. Heat fluxes are presented in kW/m². Wall surface temperatures and heat fluxes are reported with 60-s averaging to assist with interpretation of results. Oxygen, carbon dioxide, carbon monoxide, and hydrogen concentrations are presented in vol%. Total hydrocarbon concentration is presented in ppm. The smoke detector data in the supplied tables is presented as boolean data and the obscuration data is presented as the ratio of the beam signal received by the photocell to the initial clear beam signal.

### 2.3 Information

The Information (Info) directory contains a subdirectory for each of the experiments. Each subdirectory contains Microsoft Word documents that include notes transcribed during each of the experiments. These notes indicate significant events and observations that may or may not be represented in the Events CSVs in the Data directory.

## 3 Experimental Design, Materials and Methods

### 3.1 Experimental design, materials and methods

All experiments described here were conducted at the UL Large Scale Fire Test Facility in Northbrook, Illinois, US. A full report is available with additional detail, insights, and conclusions as Ref. [2]. The test facility has a floor area of 36 m by 36 m (118 ft x 118 ft) with a 14.6 m (48 ft) ceiling. The exhaust system operated with a volumetric flow rate of 420 m^3^/min (14800 cfm). With this low rate of air exchange (approximately one full air change every 45 min), the flow boundary condition for the experiments was considered quiescent and all spaces within the structures were initially quiescent.

### 3.2 Cell mock-up

Thirty 18650 form factor LIB cells were packaged into an acrylonitrile butadiene styrene (ABS) container in the configuration shown in Fig. 2. The 18650 cells were charged to 100% state-of-charge. The cells were not electrically connected to each other. A single cell in the center of the array was wrapped with a flexible film heater and heated at a rate of 6°C/min to initiate thermal runaway. This test was conducted in open air under a fume hood that was used to collect all effluent. The heat release rate was measured with oxygen consumption calorimetry and the yields of various gases were also measured from the collected effluent. The same procedure was repeated in an 82 L pressure vessel in a nitrogen atmosphere to collect all flammable gaseous components for quantification and characterization. The summary of results of the characterization from the pressure vessel experiment is presented in Table 1.

**Table 1:** Mock-up cell thermal runaway properties [2].

Property	Measurement
Cell vent temperature	130°C (266°F)
Thermal runaway temperature	204°C (399°F)
Gas volume	213 L (7.5 ft³)
Gas composition	36.2 vol% carbon monoxide 22.1 vol% carbon dioxide 31.7 vol% hydrogen 10.0 vol% hydrocarbons Hydrocarbon breakdown 7.4 vol% methane 0.92 vol% ethylene 0.61 vol% ethane 0.22 vol% propylene 0.04 vol% propane 0.07 vol% C4-hydrocarbons 0.24 vol% benzene 0.03 vol% toluene 0.38 vol% dimethyl carbonate
Gas LFL	8.9 vol%
Gas UFL	40 vol%
Gas Pmax	93 psig (641 kPa)
Gas burning velocity	35 cm/s (14 in/s)

### 3.3 Energy storage system mock-up

Experiments consisted of forcing thermal runaway propagation in a simulated LIB ESS. A single standard shipping container was used to construct the simulated ESS for all the experiments.

#### 3.3.1 ISO container

The simulated ESS was constructed in a standard 6.06 m (20 ft) International Organization for Standardization (ISO) shipping container. The standard exterior dimensions of such a shipping container are 2.43 m (8 ft) wide, 2.59 m (8.5 ft) high, and 6.06 m (20 ft) long. The measured internal volume of the container was 33.1 m³ (1169 ft³).

**Fig. 1:**
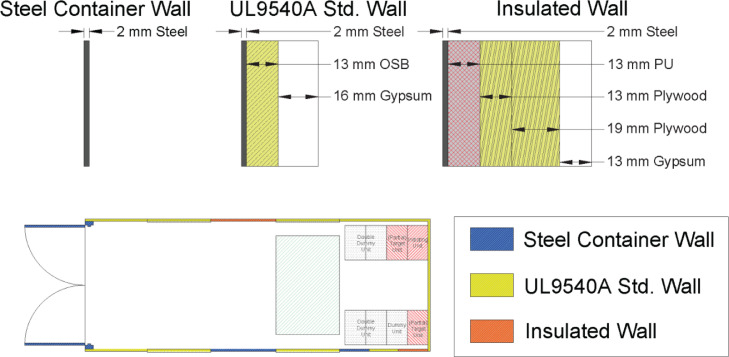
Wall construction details and container schematic.

Three different types of wall construction were built up over the container walls, as detailed in Fig. 1. These included the bare steel wall, the standard wall configuration specified in the UL9540A standard test method [1], and an insulated wall configuration. The different wall constructions were designed to enable comparison of internal and external temperature measurements with thermal imaging, and the associated impact on fire service incident size-up considerations. The ceiling construction was identical to the UL 9540A wall and the floor of the ISO container was covered with 0.013 m (0.5 in) thick cement board.

Five vents were installed on the container for relief of pressure developed by deflagration. Two of these vents were located on each of the long sides of the container and one was located on the roof. The size, position, and quantity of vents were determined using NFPA 68, Standard on Explosion Protection by Deflagration Venting. Each vent was 1.12 m (44 in) by 1.75 m (69 in) with a static activation pressure of 3.5 kPa ± 1.7 kPa (0.5 psig ± 0.25 psig).

#### 3.3.2 Suppression systems

A clean agent suppression system was installed in the ISO container for use in Experiment 2. The system delivered a quantity of Novec 1230 for an 8.3 vol% concentration. A Fike Model #80-124-125-X discharge nozzle was located at the geometric center of the ceiling of the ISO container and was connected to the clean agent reservoir via 1-1/4 in schedule 40 steel piping. One square positive pressure relief vent and one square negative pressure relief vent were installed through the roof of the ISO container. Each vent had an area of 0.093 m² (1 ft²). The vents were installed to relieve positive and negative pressures imposed by discharging the clean agent system to prevent damage to the relatively low static activation pressure deflagration vent panels. The actuation pressure of the 0.07 kPa (0.01 psig) for the positive pressure vent and 0.2 kPa (0.03 psig) for the negative pressure vent.

A water suppression system was included in the ISO container to simulate automatic fire sprinklers attached to a dry pipe system that may be installed in a LIB ESS. The system consisted of four open Spraying Systems Fulljet 35WSQ nozzles with a wide square spray pattern (ranging from 102° to 110°). The nozzles were positioned above the ESS unit racks such that the design density of water delivery was 20.8 mm/min (0.5 gpm/ft²) at the top of the ESS unit racks.

#### 3.3.3 Energy storage system units

Inside the ISO container, the mock-up ESS was comprised of three different configurations: an initiating unit, two target units, and three dummy units. The initiating unit was filled with cells to its designed capacity and was used to create a condition of cell-to-cell propagating thermal runaway. Target units, partially filled with cells, were placed adjacent to the initiating unit to enable the potential for unit-to-unit propagation of thermal runaway. Dummy units were used as ESS system mock-up visual aids.

**Fig. 2:**
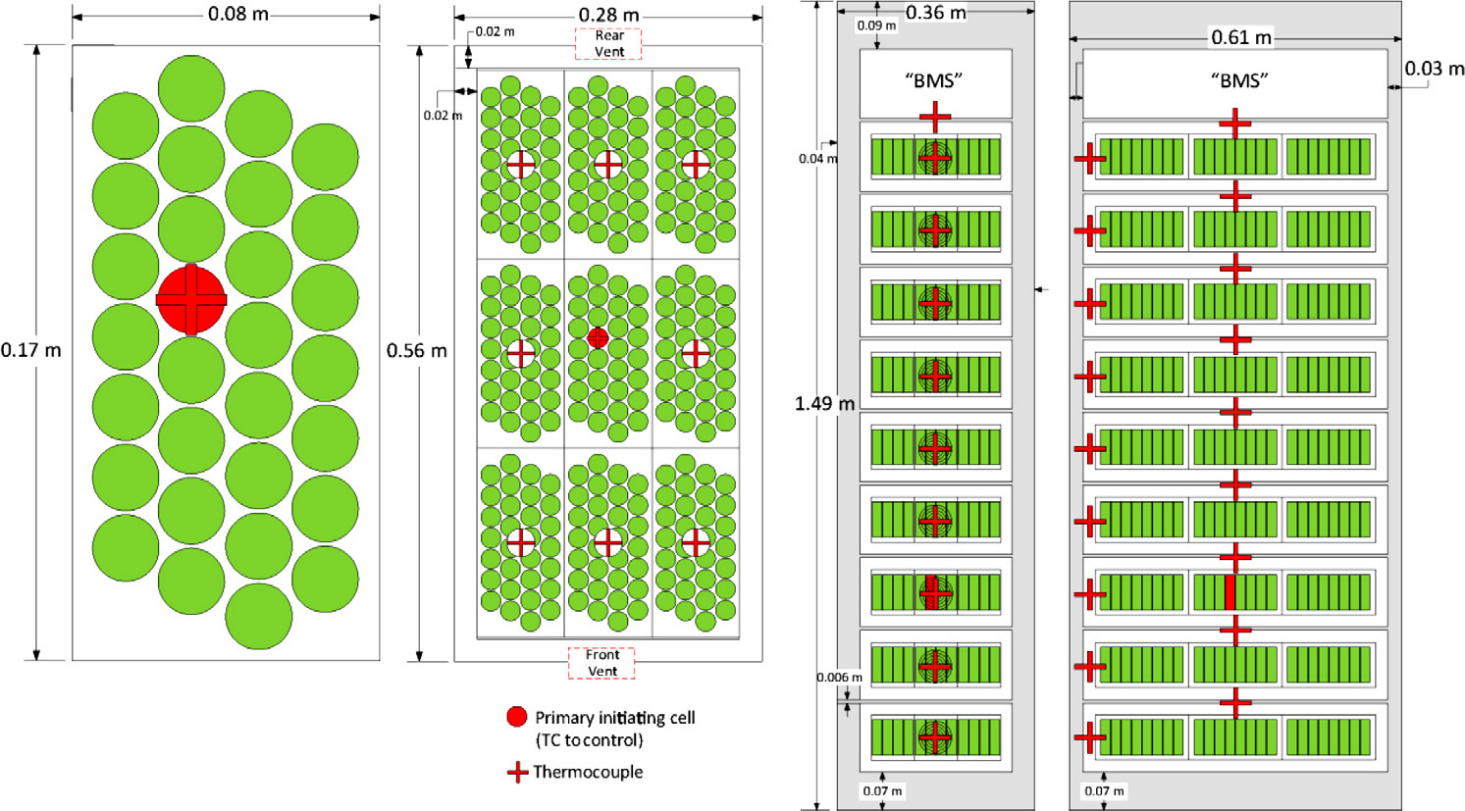
Schematics for the initiating mock-up cell, initiating module, and initiating unit rack.

The initiating unit was comprised of nine full mock-up modules oriented in a single vertical column. Full mock-up modules were comprised of nine mock-up cells oriented in a horizontal 3 × 3 grid. Each mock-up cell held thirty 18650 form factor LIB cells charged to 100% state-of-charge. Each of the 18650 cells was electrically isolated from all other cells. The geometry and layouts of the initiating mock-up cell, initiating module, and the initiating unit rack are displayed in Fig. 2. The typical mock-up cell was characterized in cell-level thermal runaway experiments conducted according to UL 9540A [1] to determine the properties displayed in Table 1.

The mock-up cell and mock-up module enclosures were composed of ABS and incorporated vent holes in the center of the front and back faces to simulate similar vents in typical ESS components. The unit racks were constructed with 90 degree steel angle stock, sheet steel panels, and perforated steel shelves to support each mock-up module. The front of the Initiating unit was open and the back and right side of the Initiating Unit was covered by uninterrupted steel sheets. The left side (adjacent to the left target unit rack) was covered with expanded steel sheets. The openings in the expanded steel enabled the communication of hot gases from the initiating unit to the left target unit during testing.

**Fig. 3:**
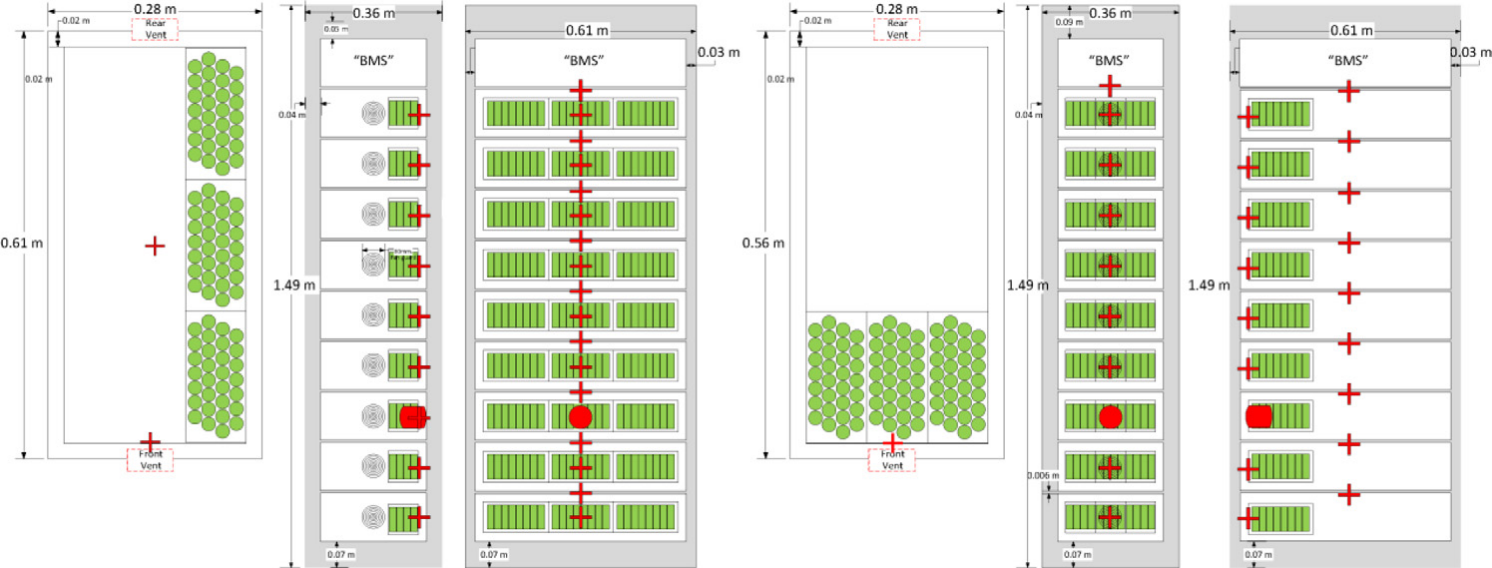
Schematics of modules and unit racks for the left and front target units.

The target units were positioned to the side and across the 0.89 m (35 in) wide aisle in the front of the initiating unit rack. Construction of the target unit racks was consistent with the initiating unit rack except each target unit was loaded with one-third the mock-up cells of the initiating unit rack. The mock-up cells were positioned in each target unit closest to the initiating unit rack. The geometry and layout of the target unit racks is displayed in Fig. 3. Dummy unit racks with no cells were installed adjacent to the target unit racks to provide realistic obstructions and installation conditions. There was no gap in the side-to-side spacing of the unit racks and no gap on the side of the initiating rack adjacent to the container wall. There was a 0.076 m (3 in) gap between the backs of the unit racks and the container walls.

### 3.4 Instrumentation

**Fig. 4:**
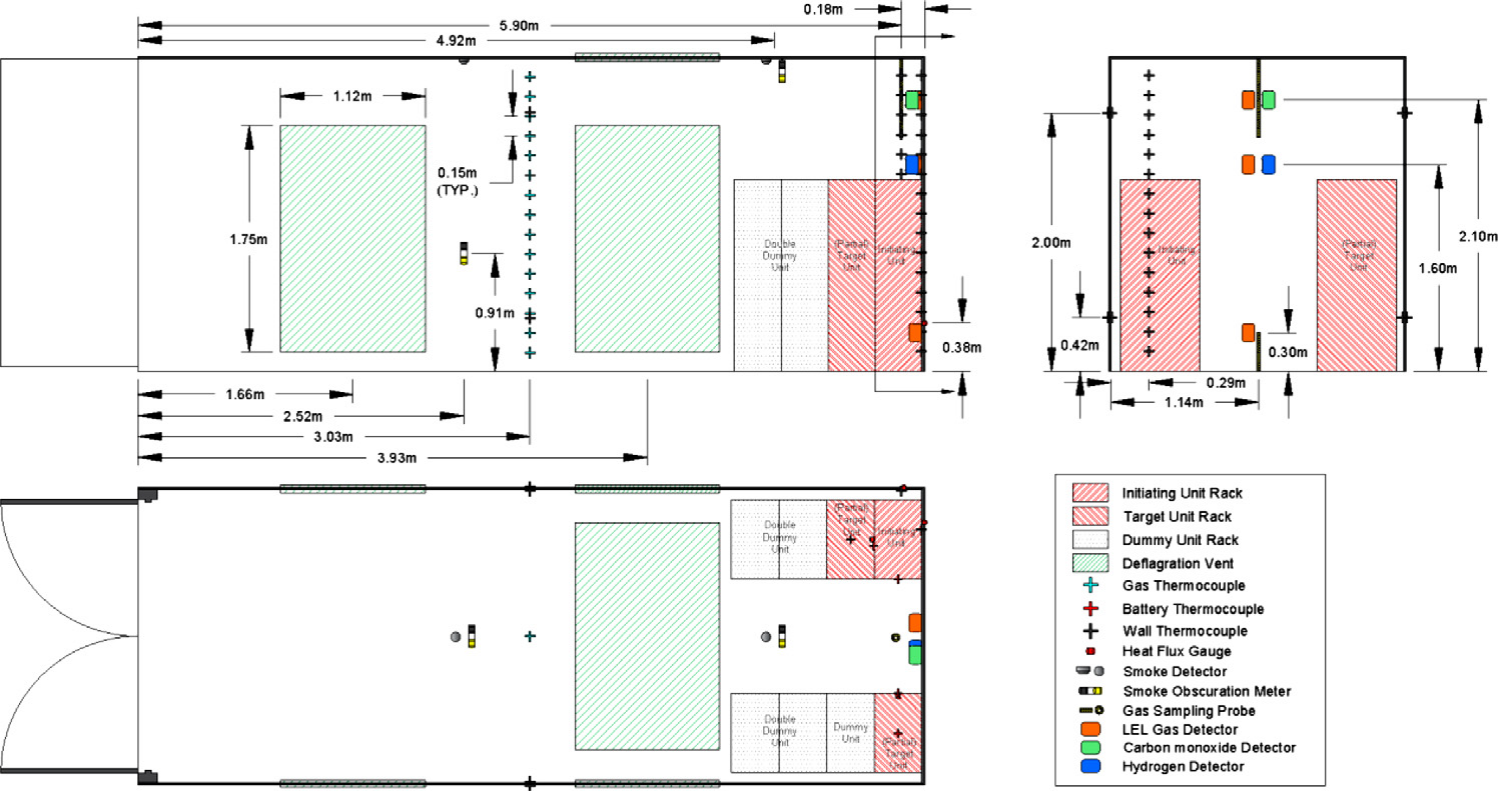
Instrumentation Schematic for All Experiments

The instrumentation layout is illustrated in Fig. 4. Instrumentation was positioned to quantify thermal conditions throughout the container, measure gas concentrations generated, and characterize smoke conditions due to thermal runaway.

Container gas and wall surface temperatures were monitored using three thermocouple arrays and additional single point thermocouple measurements. Array thermocouples were 24 American Wire Gauge (AWG) Type K and single thermocouples were 30 AWG Type K.

One thermocouple array was attached to each instrumented wall, to the side and rear of the initiating unit rack. The arrays were centered on the initiating unit rack with a thermocouple located every vertical 0.15 m (6 in) from floor to ceiling. The walls instrumented with the thermocouple arrays were painted flat black to enhance and control emissivity. Additional pairs of thermocouples were installed coaxially through the thickness of the wall in four locations on corresponding internal and external surfaces of the container.

An additional thermocouple array was installed in the center of the container to measure gas temperatures. Thermocouples were installed every 0.15 m (6 in) vertically from floor to ceiling. Two 24 AWG Type K thermocouples were installed in each module of the initiating unit and target units; one was installed on the underside of the lid in the center of the module, and one was installed inside the front vent opening of the module (Fig. 2). Both thermocouples were used to determine whether thermal runaway had occurred within the module.

One Schmidt-Boelter heat flux gauge was installed flush with the surface of each instrumented wall at 0.4 m (15.7 in), the height of the initiating module, to measure the heat flux incident from the initiating unit rack to the adjacent wall. Heat flux gauges were also installed in each target unit at the same elevation as the initiating module, oriented such that they were directed at the initiating unit rack as shown in Fig. 3.

A combination of analytical instruments and common industrial gas detectors were used to characterize the gas composition inside the container. Gas samples near the ceiling and floor were extracted from the container and transported by heated lines to analytical instruments. The sample taken near the ceiling was analyzed for oxygen, carbon monoxide, carbon dioxide, hydrogen, and total hydrocarbon concentrations. The sample taken near the floor was analyzed for total hydrocarbon concentrations to measure the stratification of lighter and heavier than air hydrocarbons.

Common industrial gas detectors included hydrogen detectors, carbon monoxide detectors, and combustible gas detectors were also installed in the container. The industrial gas detectors were selected to be representative of the types that may be installed in ESS installations; the data from these meters is intended to represent what may be available for hazard assessment if the signals are remotely monitored. All three detector types were mounted on the wall between the initiating unit rack and front target unit rack. Three combustible gas detectors were utilized to compare with total hydrocarbon measurements of stratification in the gas layer.

Two commercially available smoke detectors were installed along the centerline of the container and evenly spaced at one-third of the lengths of the container. The smoke detector closer to the initiating unit was labelled “Near” and the second was labelled “Far.” Both detectors were combination photoelectric/ionization. Smoke detectors were incorporated because of their common application as initiating devices for fire alarm and fire suppression systems.

Smoke obscuration was measured at the ceiling adjacent to the smoke detector near the unit racks and 0.91 m (3 ft) above the floor below the “Far” smoke detector. Obscuration was measured with a white light source and photocell receiver with a 0.91 m (3 ft) path between them.

## 4 Experimental

Prior to each test, each analytical gas instrument was field calibrated. New smoke detectors and commercial gas detectors were installed for each test. Each test began by energizing a flexible film heater wrapped around an individual 18650 cell in the initiating mock-up cell. The instrumented 18650 cell was heated at a rate of 6°C/min to initiate thermal runaway. Heating continued at this rate until thermal runaway was observed, at which point the heater was de-energized.

Thermal runaway behavior was confirmed by a rapid increase in cell surface temperature exceeding 10°C/s (50°F/s) to a maximum temperature in excess of 500°C (932°F) for the 18650 cell at 100% state of charge. In contrast, cell venting was marked by temperature fluctuation on the cell surface of less than 10°C (50°F) within five seconds as the cell safety vent operates and relieves electrolyte vapor pressure within the cell case.

Following the initial thermal runaway event, thermal runaway propagation behavior of the ESS was monitored until actions were required to activate suppression systems. In Experiment 1, no suppression systems were utilized. In Experiment 2, the Novec 1230 system was discharged with a solenoid valve upon activation of both smoke detectors installed in the container. In Experiment 3, the water suppression system was activated 30 seconds after actuation of a 74°C (165°F) standard response sprinkler link.

After the deployment of suppression systems, as applicable, the behavior of the ESS was observed until test termination. While observing test progression, propagation of thermal runaway to additional modules in the initiating unit and target unit racks was monitored. Thermal runaway behavior in these modules was marked by an immediate temperature increase of more than 400°C (752°F) and sustained temperatures above 300°C (572°F). When thermal runaway activity subsided or module temperatures began decreasing, test termination procedures were initiated.

To begin test termination procedures, a carbon dioxide system was used to mitigate potential deflagration hazards. The system discharged 41 kg (90 lb) of carbon dioxide to develop a concentration of 62 vol% carbon dioxide before the doors were allowed to be opened. The operation of the system was verified before conducting the test series. Following the deployment of the carbon dioxide system, the container doors were opened remotely utilizing electrically operated winches. Once the deflagration hazard was mitigated, manual extinguishment, overhaul, and disposal were conducted. This procedure was utilized for all three tests.

## Ethics Statements

This project does not involve human subjects, animal experiments, or data collected from social media platforms.

## CRediT authorship contribution statement

**Mark McKinnon:** Writing – original draft, Data curation. **Adam Barowy:** Conceptualization, Methodology, Investigation, Writing – review & editing. **Alexandra Schraiber:** Methodology, Investigation, Writing – review & editing. **Jack Regan:** Methodology, Investigation, Data curation.

## Declaration of Competing Interest

The authors declare that they have no known competing financial interests or personal relationships that could have appeared to influence the work reported in this paper.

The authors declare the following financial interests/personal relationships which may be considered as potential competing interests:

## Data Availability

2020 UL 9540A Installation-Level Test Experimental Data (Original data) (Zenodo).
